# Exploring Glyoxalase Strategies for Managing Sugar-Induced Chronic Diseases

**DOI:** 10.3390/life15050794

**Published:** 2025-05-16

**Authors:** Alejandro Gugliucci

**Affiliations:** Glycation, Oxidation and Disease Laboratory, Touro University California, Vallejo, CA 94592, USA; alejandro.gugliucci@gmail.com

**Keywords:** methylglyoxal, glyoxalases, fructose, de novo lipogenesis, dyslipidemia, metabolic dysfunction-associated steatotic liver disease, glycation, ketohehokinase c, metabolic syndrome

## Abstract

The liver’s crucial role in methylglyoxal (MG) metabolism is frequently overlooked in the literature. We present a perspective that enhances the current understanding of the role of methylglyoxal (MG) and the glyoxalase cycle in the pathogenesis of insulin resistance and obesity, ultimately leading to type 2 diabetes mellitus (DM) and cardiovascular disease (CVD). Fructose may be a significant substrate contributing, particularly in contemporary times, to the flux of trioses in the liver, accounting for a substantial portion of MG production. The steady-state concentration of MG—and the subsequent modification of proteins—would then be determined by the flux of trioses, their utilization in lipogenesis, and their decomposition into MG, which is further converted into D-lactate by glyoxalase enzymes GLO1 and GLO2. Consequently, enhancing the activity and/or expression of GLO1 could potentially mitigate the adverse effects of fructose in the liver. Additional research and validation are required to confirm these biological pathways. These arguments are in favor of further research into safe and efficient ways to activate the glyoxalase pathway to lessen the negative effects of fructose metabolism that lead to insulin resistance (IR) and its related repercussions.

## 1. Introduction

The human glyoxalase family of proteins includes six members with significant variations in enzymatic activity, protein structure, and tissue specificity [1,2,3]. This family is primarily involved in detoxifying reactive dicarbonyls, of which methylglyoxal (MG) is the most significant. Due to their central role, these highly conserved enzymes are important for maintaining homeostasis and preventing the development of metabolic diseases like diabetes and cancer [1,2,3,4,5,6,7,8]. MG is traditionally associated with glycolysis and serves as an indicator of unbalanced flux between dihydroxyacetone phosphate (DHAP) and glyceraldehyde-3-phosphate (G3P). MG stems from a metabolic node, the trioses, shared with gluconeogenesis and glycerogenesis [2,3,9,10,11,12,13]. The latter ultimately contributes to triacylglycerol (TG) synthesis. Due to its protein-modifying abilities, MG has been implicated in obesity and insulin resistance (IR), prompting the development of activators that enhance the expression of glyoxalase 1 (GLO1), its primary catabolic enzyme [1,2]. Although the initial results are modest, early trials have demonstrated the potential utility of targeting the glyoxalase pathway for managing the increasing prevalence of metabolic syndrome, obesity, and diabetes. The literature primarily focuses on glycolysis in a somewhat non-specific way, as a source of MG, often overlooking the liver’s central role in MG metabolism. We present an alternative perspective on this significant topic by highlighting two areas that warrant further research. Firstly, the metabolism of fructose should be considered an important source of MG. It involves three distinct enzymes different from those in glycolysis and is poorly regulated by feedback mechanisms and thus prone to overflow. We examine how fructose metabolism may be the primary source of MG in the liver, due to frequent fructose surges provided by the Western diet and the fact that over 80% of fructose metabolism occurs in the liver during first-pass uptake. These points support ongoing efforts to identify safe and effective methods to stimulate the glyoxalase pathway to mitigate the harmful effects of fructose metabolism that contribute to IR and its associated consequences. Thus, this article will provide an overview of methylglyoxal (MG) metabolism and glyoxalases and will then focus on key aspects of fructose metabolism in relation to IR and its associated conditions, including dyslipidemia, diabetes, and cardiovascular disease (CVD). Subsequently, we will examine hepatic glucose and fructose metabolism in parallel, to highlight their differences as well as their intricate and synergic interactions. We present our evidence suggesting that MG metabolism (with D-lactate measurement as a proxy) can be influenced by fructose restriction, suggesting that activation of glyoxalase may be beneficial in situations where dietary fructose reduction is not feasible.

## 2. Methylglyoxal: Sources and Deleterious Effects

### 2.1. MG Is the Primary Source of Advanced Glycation End-Products In Vivo

**Figure 1:**
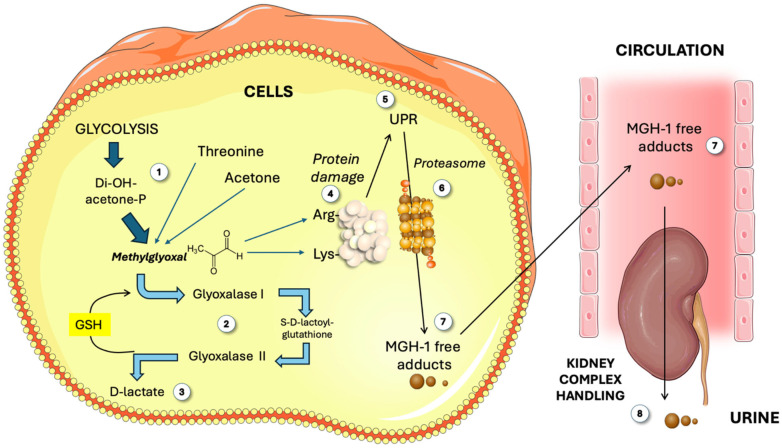
**Overview of methylglyoxal (MG) metabolism**. The classic main source of MG is glycolysis (1), as well as threonine and acetone. MG is catabolized (2) by Glo1 and 2 to render D-lactate (3). Free MG attacks Arg and Lys residues in proteins (4). Damaged proteins elicit the unfolded protein response (5) and proteasome activity (6), yielding MGH-1 free adducts (7) which circulate and are finally disposed of by the kidney (8). GSH: reduced glutathione; MG: methylglyoxal; MGH-1: methylglyoxal-hydroimidazolone 1; UPR: unfolded protein response. This figure was partly generated using Servier Medical Art, provided by Servier, licensed under a Creative Commons Attribution 3.0 unported license.

MG has traditionally been considered to be primarily generated in proportion to glycolytic flux through the spontaneous degradation of the triose phosphates dihydroxyacetone phosphate (DHAP) and glyceraldehyde 3-phosphate (G3P) at a rate of 0.1–1% of triose flux [4,5,7,8]. Furthermore, other minor metabolic pathways, such as glycoxidation and threonine and acetone catabolism, also contribute to the MG pool, as depicted in Figure 1. Under physiological conditions, it is estimated that less than 10% of MG exists as a free aldehyde, with the majority being bound to macromolecules, particularly glutathione [4,11,12,14]. Plasma concentrations of MG in humans range from 50 to 250 nM, while intracellular concentrations are maintained between 2 and 10 μM, depending on the physiological context and cell type. MG has been extensively studied in relation to obesity, IR, and diabetes, mainly based on the fact that its formation is highly proportional to glycolytic flux [2,11,12]. The proportionality stems from triose flux, which, as we shall discuss, may rather depend largely on fructose flux in the liver. Small amounts of MG seem to be necessary for proper organismal function, such as the “hormetic” response to acute stress [10]. However, MG accumulation can lead to cellular dysfunction because it is several orders of magnitude more reactive than glucose in the non-enzymatic modification of proteins and DNA. Free MG primarily induces damage to proteins through rapid interactions with thiols, lysine residues, and arginine residues, as shown in Figure 1. MG modification of lysine residues results in the formation of several irreversible advanced glycation end-products, including carboxyethyl lysine (CEL), lysine-lysine crosslinks (MOLD), and lysine-arginine protein crosslinks (MODIC). Nevertheless, arginine remains the principal amino acid target for MG-induced irreversible modifications, with the predominant AGE product being methyglyoxal-hydoimidazolone 1 (MG-H1). MG-H1 is identified as the most prevalent circulating AGE, whereas carboxyethyl arginine (CEA) is recognized as the primary cellular MG adduct [4,11].

### 2.2. Effects of MG Adducts

MG modification increases the molecular volume of arginine residues by 18%, making them more hydrophobic. Arginine residues are often found in enzyme catalytic domains, so MG modification can alter protein function [4,11]. Beyond active sites, there are other structural considerations. Indeed, polar residues like arginine on protein surfaces enhance solubility in the cytosol. MG-H1 formation eliminates its polarity by loss of a positive charge. This affects interactions with the environment, translating into shifts in the secondary structure. Consequently, the increased hydrophobicity due to MG-H1 signals misfolded or damaged proteins, activating the unfolded protein response (UPR). The UPR activates the ubiquitination and degradation of MG-modified proteins via the 26S proteasome [9,12,15], as shown in Figure 1. However, modified amino acids remain unchanged after protein degradation and are cleared from the cell as AGE free adducts. The modified amino acids in circulation, whether from cellular degradation or direct modification of circulating peptides, are excreted by the kidney in a complex management process, as depicted in Figure 1 [16].

### 2.3. MG, MetS, Diabetes, and Obesity

MG is known to predict diabetic complications, including neuropathy, nephropathy, and retinopathy. Cell culture and animal models, including those of our group, suggest MG drives systemic metabolic dysfunction, contributing to diabetes development [4,6,7,8,9,10,17]. Nonetheless, in humans, data on MG levels in obesity and their link to IR are limited, partly due to the absence of a reliable commercial assay for MG, the complexity of the LC-MS/MS methodology, and the lack of high-purity MG standards. Although current evidence is limited, data suggest MG accumulation may drive obesity-related metabolic dysfunction. For instance, plasma MG levels are ~37% higher in overweight individuals and ~85% higher in obese participants [1,2,11,13]. The exact mechanism, primary affected tissues, and responsible factors are unknown. Little attention has been paid to the liver in these matters; we will bring this issue to the limelight.

There is, henceforth, substantial evidence indicating that increased dicarbonyl stress, induced by the accumulation of MG, contributes to metabolic dysregulation. This phenomenon is likely influenced by one or both of the following factors:

- Elevated metabolic flux at baseline and post-feeding due to IR and consequences of poor handling of glucose, to which, as we propose, fructose metabolism should be added.
- Reduced capacity for MG detoxification due to diminished expression or activity of glyoxalases, key enzymes in MG disposal.

## 3. Glyoxalases

In view of the toxicity of MG (and glyoxal secondarily), nature has evolved a set of highly conserved enzymes to detoxify it. The human glyoxalase protein family comprises six members that exhibit significant variations in enzymatic activity, protein structure, and tissue specificity. Their primary function is the detoxification of reactive dicarbonyls [18,19,20]. Although adapted to a wide array of chemical reactions, certain fundamental characteristics have been conserved within the glyoxalase gene family. Despite the limited sequence homology, all members share similar protein fold domains, specifically the glyoxalase domain, which is essential for their metal–ligand binding capabilities. These enzymes likely evolved to accommodate diverse divalent metal ions, contributing to their broad substrate profile [2,18,19,21,22].

Among the six main classes of glyoxalases, GLO1 and 2 specifically deal with MG.

### 3.1. Glyoxalase 1

GLO1 is found in humans and across nature, highlighting its vital role in metabolism and aldehyde detoxification. The protein functions in the cytosol as a homodimeric Zn^2+^-dependent metalloenzyme. Its active site, located at the dimer interface, binds Zn^2+^ with His127 and Glu173 from one subunit and Gln34 and Glu100 from the other [18,22,23]. The human GLO1 gene is located at chromosome 6p21.2 and consists of six exons that encode the 21 kDa GLO1 protein. The promoter region of GLO1 contains several regulatory elements, including antioxidant response element (ARE), metal response element, and insulin response element binding sites for several transcription factors, which include nuclear transcription factor-κ. Despite the presence of these regulatory elements, there is ongoing debate regarding the transcriptional regulation and inducibility of GLO1. Although increased expression of GLO1 has been observed following activation of the antioxidant stress response, conflicting reports exist [1,18,21].

### 3.2. Glyoxalase 2

Interestingly, despite sharing the name glyoxalase, glyoxalase 2 (GLO2) is not part of the glyoxalase gene family. Located at Chr 16p13.3, GLO2 consists of 10 exons encoding a 29 kDa protein. It is the rate-limiting enzyme for the glyoxalase cycle and is the only known enzyme that hydrolyzes LGSH in humans. Much remains to be studied about the role of GLO2 in human disease [18,19,21,22,24].

### 3.3. Glyoxalase Cycle

As depicted in Figure 1, GLO1 primarily reacts with MG, generating s-D-lactoyl glutathione (LGSH) which is a substrate for GLO2 in the glyoxalase cycle [2,10,19,21,24,25]. GLO2 then hydrolyzes LGSH to produce D-lactate and recycle GSH. Besides MG, GLO1 also acts on other α-oxoaldehydes like glyoxal, phenylglyoxal, and 3-deoxyglucosone. Note that GLO1 requires the reduced form of the antioxidant glutathione to supply the sulfhydryl group needed for the hydration of MG to produce D-lactate. The pool of this antioxidant is diminished by a diet high in processed foods and sweets, such as the Western diet [26,27,28,29,30]. Moreover, reduced glutathione in this cycle then necessitates reducing power sourced from NADPH (e.g., pentose phosphate cycle). Oxidative stress consuming NADPH may conspire against proper GLO1 and 2 kinetics. GLO1 and 2 play a crucial role in maintaining homeostasis and are essential for preventing the onset of metabolic diseases such as diabetes and cancer. For more extensive details, readers should refer to other specific contributions in this Special Issue.

## 4. Fructose: An Inadequately Researched Contributor to Hepatic MG

### 4.1. Fructose and Metabolic Disease

Fructose and its impact on metabolic health have been a topic of considerable interest, particularly in the context of IR and its associated conditions, MetS, obesity, diabetes, and related cardiometabolic disorders such as metabolic-associated steatotic liver disease (MASLD), previously known as non-alcoholic fatty liver disease (NAFLD) [29,31,32,33,34,35]. The incidence of these disorders has been rising, influenced by a complex interplay of hereditary and environmental factors, including nutrition and physical exercise. Over the past 50 years, the availability of sugar, especially in the form of sweetened beverages, has increased due to improved industrial processes and corn subsidies. This surge in sugar availability is associated with a rise in the prevalence of cardiometabolic disorders [31,32,34,35,36,37].

Sugar is commonly added to beverages and food products in the form of sucrose or high-fructose corn syrup (HFCS), both of which are rich sources of glucose and fructose, with a fructose content of at least 50%, but more in HFCS. In the 1970s [38,39,40,41], studies demonstrated that diets high in fructose could promote hypertriglyceridemia in both animals and humans more rapidly than diets containing comparable amounts of starch or glucose. Hypertriglyceridemia caused by sugar and polymeric carbohydrates was closely linked to hyperinsulinemia in both obese humans and animals. This research was mostly silenced for four decades. Indeed, some authors argue that the question of whether added sugars and naturally occurring dietary sugar contribute to the epidemic of cardiometabolic disease is still under discussion, suggesting that the issue ultimately relates to calories and obesity. A review of the funding sources for many of these studies indicates connections to the food and beverage industry, potentially introducing bias [42,43,44,45]. These publications may create uncertainties in the advancement of science and are akin to classic tactics used by the cigarette industry in the past [46,47,48]. Another point of debate is the experimental design in some studies which involved overfeeding with fructose alone, which does not accurately reflect typical human consumption: fructose is usually ingested as part of sugar or HFCS.

**Figure 2:**
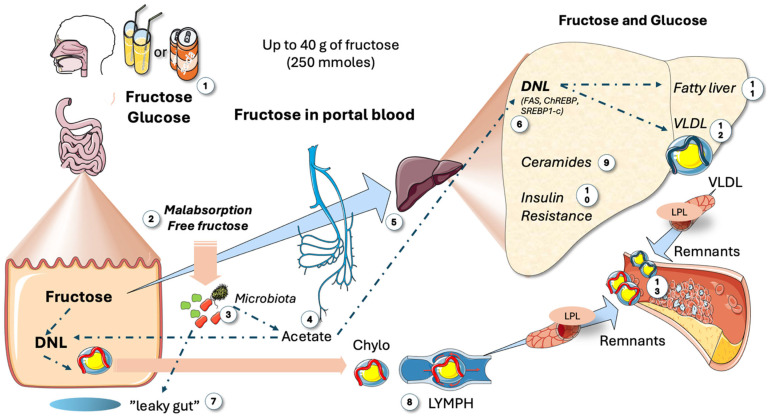
**Main metabolic impact of large intakes of fructose**. When consumption is high, as in the Western diet (1), which can amount to up to 25% of the caloric intake in USA adolescents, several deleterious reactions occur: Malabsorption of fructose (2) is frequent because glucose absorption is faster. Residual fructose feeds the microbiota (3), which produce acetate (4) that is then directed to the liver via the portal system (5) and leads to fat synthesis by de novo lipogenesis (DNL) (6). Fructose supports the development of microbiota, which may contribute to leaky gut (7). This condition allows lipopolisaccharides from bacteria to reach the liver, potentially causing inflammatory effects and increasing the production of chylomicrons (8). A saturated enteral barrier allows for the escape of much of the absorbed fructose into the portal system (5), inundating the liver and producing an array of deleterious reactions, including the production of specific ceramides (9) that result in IR (10) and either fatty liver (11) or TRL dyslipidemia (12). Together with enteral chylomicrons (8), VLDLs generate remnants (13) which are more atherogenic than LDLs. DNL: de novo lipogenesis; IR: insulin resistance; VLDLs: very low-density lipoproteins; LDLs: low-density lipoproteins; LPL: lipoprotein lipase. This figure was partly generated using Servier Medical Art, provided by Servier, licensed under a Creative Commons Attribution 3.0 unported license.

As we depict in an overview in Figure 2, sugar in food, and especially in liquid form, has significant metabolic consequences that are more severe when consumed on an empty stomach. The most serious effects occur with large, rapid, and chronic intake [34,35,49,50,51,52]. The American Heart Association recommends that both adults and children consume less than 10% (preferably less than 5%) of their energy needs from free sugar to reduce the risk of obesity and metabolic disorders [31,53]. This equates to roughly 330 mL of soda or fruit juice. In the USA, it is common for sugar consumption to account for 25% of daily energy intake, particularly among children and adolescents [54,55]. Sugar-sweetened beverages (SSBs) are the primary source of added sugar and fructose intake in both children and adults, and their consumption is consistently associated with an increased risk of cardiometabolic diseases, including elevated cardiovascular mortality related to poor diet. Free sugars include the mono- and disaccharides naturally found in honey, syrups, fruit juices, and fruit juice concentrates, as well as those that are added to foods and beverages [56].

Recent studies across Europe, Latin America, and the USA have found that average sugar intakes in many of these regions exceed the recommended levels [56,57,58,59,60,61]. Consequently, several countries are considering or have already implemented measures to reduce sugar consumption, such as better food labeling or taxes on sweetened foods. Most natural fruit juices contain fructose concentrations akin to HFCS-55-sweetened beverages. For example, orange juice typically contains 51–57 g/L of total fructose (including free fructose and fructose from sucrose), accounting for 52–54% of its total sugar content. A glass of orange juice comes from four to five oranges. Typically, individuals do not consume this many oranges at once, and consuming the whole fruit provides a significant amount of fiber, which slows the absorption of free sugars [62,63,64,65,66,67]. Apple juice has a fructose-to-glucose ratio of 2:1. This is referred to as excess free fructose (EFF). A glass of 100% apple juice contains 8 to 9 g of EFF. Excess free fructose has negative impacts on the microbiota and intestinal health, in addition to its effects on the liver (Figure 2).

Before discussing the specifics of fructose vs. glucose hepatic metabolism, Figure 2 serves as a bird’s eye view of the key facts, as reviewed in [68,69]. Small doses of sugar, such as in fruit, are metabolized by the intestines into glucose or lactate for liver circulation. However, as depicted in the figure, large consumption, akin to 25% of the caloric intake in some American teenagers, leads to harmful effects: malabsorption of fructose and the resulting residual fructose feeds the microbiota, producing acetate, which is then directed to the liver and participates in fat synthesis. Fructose also promotes unwanted microbiota growth, causing leaky gut and inflammatory effects on the liver [70]. Data show that DNL even occurs in the intestines, increasing chylomicron secretion. Saturated enteral barriers let absorbed fructose flood the portal system, leading to increased activation of carbohydrate-responsive element-binding protein (ChREBP) and sterol regulatory element binding protein 1c (SREBP1c), stimulating ceramide synthesis, de novo lipogenesis (DNL), IR, fatty liver, and/or dyslipidemia [32,33,69].

### 4.2. Fructose Metabolism vs. Glycolysis

#### 4.2.1. Fructose as a Source of MG

**Figure 3:**
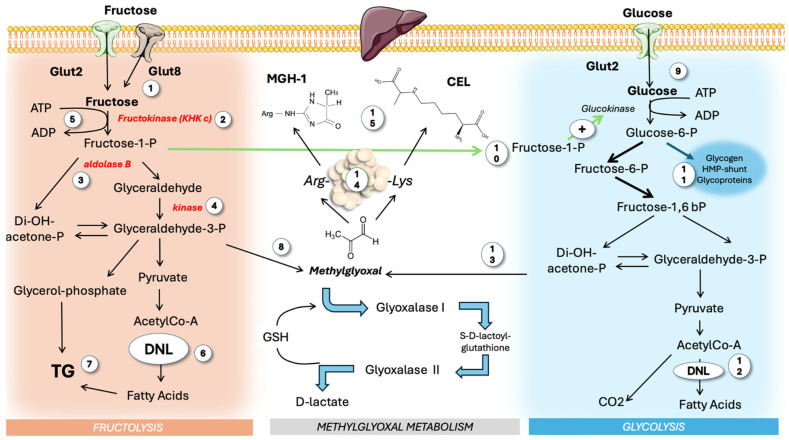
**Hepatic fructose and glucose metabolism: differences and synergies**. The majority of dietary fructose is consumed as sugar or HFCS, carrying similar masses of fructose and glucose, which reach the liver simultaneously. Fructose (1) is acted upon by three unique enzymes different from those of glycolysis: KHK c (2), aldolase b (3), and glyceraldehyde kinase (4). KHK c uses ATP (5) in a non-regulated fashion that may lead to ATP depletion. Fructose stimulates DNL (6) and TG synthesis (7). Trioses are also sources of MG (8). Glucose (9) undergoes classic feedback-regulated glycolysis, albeit it is strongly stimulated by F-1-P from fructose metabolism (10). Glucose-6-P also shunts to other pathways and (11) adds to the pool of DNL (12), potentiating fructose action. Trioses from glucose (13) are sources of MG as well. Free MG then modifies proteins (14), leading to MGH-1 and CEL (15). MG: methylglyoxal; MGH-1: methylglyoxal-hydroimidazolone 1; KHK c: ketohexokinase c; CEL: carboxy ethyl lysine; DNL: de novo lipogenesis. This figure was partly generated using Servier Medical Art, provided by Servier, licensed under a Creative Commons Attribution 3.0 unported license.

After reviewing fructose metabolism in parallel with glycolysis, it becomes clear that a key source of hepatic MG is fructose. In Figure 3, we compare both metabolisms as well as their interactions and their roles as generators of MG. Fructose is primarily metabolized in the liver (more than 80% of the intake) using three unique enzymes, namely ketohexokinase c, aldolase B, and glycerokinase, which convert it into triose phosphates that then integrate with cellular pools from glycolysis and gluconeogenesis. The enzyme ketohexokinase c (KHKc), or fructokinase, initiates fructolysis by irreversibly converting fructose to fructose-1-phosphate, distinct from glycolysis or gluconeogenesis pathways [71,72,73,74,75,76]. Unlike glucose, fructose is a weak hexokinase and, even less, glucokinase substrate, and KHKc, which is always active and not inhibited by ATP, has a higher phosphorylation rate for fructose than glucokinase has for glucose. Consequently, most of the fructose entering the liver is rapidly metabolized and does not reach the systemic circulation due to KHKc’s high affinity and activity. As a corollary, a transient decrease in ATP (with a concomitant increase in AMP) is produced during hepatic surges of fructose [77,78,79,80].

#### 4.2.2. The KHKc Product Fructose-1-Phosphate Stimulates Glycolysis

The interplay between fructose and glucose metabolism in the liver underscores the role of fructose-1-phosphate (F-1-P) as a regulatory molecule that has been disrupted by modern dietary habits. The reaction catalyzed by fructokinase produces fructose-1-phosphate, which is subsequently acted upon by aldolase b, generating the three-carbon molecules dihydroxyacetone phosphate (DHAP) and glyceraldehyde (GA). A glyceraldehyde kinase generates G-3-P. Unlike glucokinase and hexokinase, which have exquisite feedback regulation, fructokinase activity remains unchecked. Furthermore, fructose consumption typically coincides with glucose intake, either as sucrose or HFCS. As illustrated in the figure, like fructose, glucose is also transported into hepatocytes via Glut 2 transporters. F-1-P acts as an allosteric mediator that enhances glucose metabolism through its effects on glucokinase (GK) and pyruvate kinase, increasing the flux of intermediates, leading to DNL and esterification with glycerol, resulting in TGs as end products (which add to the TGs stemming from glucose). Evidence from fructose-intolerant patients and animal models, which show elevated F-1-P levels but reduced fructolytic flux (yet associated with greater steatosis), supports this mechanism [81]. TGs are packaged with apoB100 in very low-density lipoproteins (VLDLs) or stored as liver fat, as shown in the figure. Overflow of the former may lead to dyslipoproteinemia, the latter to MASLD. Fructose at “catalytic” concentrations can significantly elevate hepatic glucose levels. Fructose’s ability to raise hepatic F-1-P levels, leading to the release of GK, is a key primary mechanism by which fructose drives DNL in the liver [30,82,83,84,85]. F-1-P likely evolved as an efficient means for processing scarce, intermittent dietary fructose, a mechanism now disadvantageous in contemporary diets [79,86].

#### 4.2.3. The Triose Node: Backbone of TGs and Source of MG

Fructose contributes up to 30% of the glycerol backbone of triglycerides (TGs) and acts as a potent stimulus for DNL via fructose-1-phosphate. Lastly, during insulin resistance (IR), even fructose-derived carbons can be incorporated into FA within TGs, reaching up to 30%. It is evident that the same fluxes of trioses produced by fructolysis, compounded by contributions from glycolysis, feed lipogenesis (fatty acid and TG synthesis) and MG production, creating an eventual overload of the GLO pathway, increased MG steady-state concentrations, and potential protein damage [33,49,59,86,87,88]. Increased fructolytic flow may also provide substrates and reducing equivalents to support fatty acid synthesis beyond the aforementioned pathway. It has been proposed that uric acid, produced through rapid fructose metabolism, further promotes DNL [59,86]. In fact, research has shown that the liver FA production mechanism is particularly significant in understanding the causes of dyslipidemia and MASLD.

#### 4.2.4. Evidence for the Lipogenic Activity of Fructose

Studies examining the effect of increased CHO/sugar/fructose consumption indicate that even with maintenance dietary interventions, ingesting carbohydrates, especially simple sugars in liquid form, promotes hepatic lipogenesis. Notably, studies specifically examining the effects of other hexoses (such as glucose and fructose) suggest that fructose is a more effective inducer of lipogenesis than glucose. It is important to acknowledge that DNL generates new fatty acids (FAs), while the liver also synthesizes TGs using fatty acids sourced from plasma, remnants, and storage droplets. In a nutshell, fructose stimulates liver DNL by providing carbons for FAs and glycerol for TGs. Insulin resistance integrates glucose and fructose metabolism through ChREBP, SREBP1c, and fructose-1-P [78,88,89,90,91,92,93,94,95,96,97,98,99].

Studies on fructose overfeeding and restriction, including ours, indicate that fructose contributes to the overproduction of TRL in humans. Research has focused on the effects of fructose on hepatic very low-density lipoprotein (VLDL) metabolism, which affects total plasma TG levels. Several dietary studies in both animals and humans have examined these effects. Recent interventional studies lasting from weeks to months have shown that overfeeding humans with moderate to high doses of fructose-containing sugars can negatively impact metabolic outcomes.

The addition of SSBs, constituting 10% to 25% of required energy intake, increased cardiovascular risk factors such as lipids and uric acid [93,94,95,100,101,102]. A study investigated the effects of providing overweight or obese individuals with isocaloric beverages sweetened with glucose or fructose for 10 weeks, accounting for 25% of their basal energy needs [94,95]. Both treatments resulted in similar increases in body weight; however, only fructose led to increased visceral adiposity, DNL, atherogenic dyslipidemia, and indicators of insulin resistance. Restriction studies (including our own, as shown in the next section), particularly those involving children and adolescents, provide substantial clinical evidence that dietary sugar intake, in proportions typical of Western diets, correlates with adverse metabolic health outcomes [49,88,96,97,98,99]. Depending on the study population, the level of sugar restriction, and the duration of the study, some interventions aimed at reducing SSB consumption have shown reductions in weight gain, adiposity, liver fat, and indicators of insulin resistance.

## 5. Proof of Principle: Human Evidence of the Link Between Fructose, MG, and Lipogenesis

As pointed out above, in addition to providing glycerol for TG synthesis, trioses from fructose catabolism also generate methylglyoxal. From a molecular standpoint, unstable DHAP spontaneously breaks down to produce higher MG. Consequently, elevated triose phosphates (TPs) play a crucial role as bridges in the pathways of glycolysis, gluconeogenesis, and glycero-phosphate (α-GP) synthesis. In fact, as discussed earlier, methylglyoxal, which is several orders of magnitude more reactive than glucose, has garnered more attention in recent decades for intracellular glycation. However, as we pointed out, most articles neglect the fact that a major source of MG in the liver may be fructose and not glucose, at least in people with Western diets.

**Figure 4:**
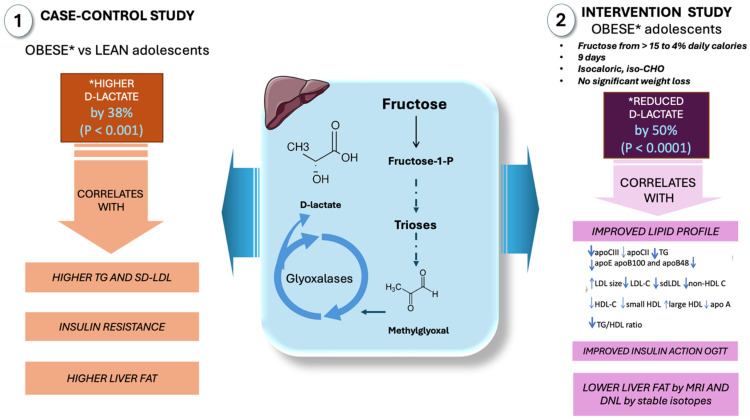
D-lactate as a surrogate marker of high MG fluxes in adolescents: case–control and intervention studies in our laboratory. In a case–control study involving obese and control adolescents, increased levels of D-lactate were observed in the obese group, correlating with insulin resistance, liver fat, and dyslipidemia. When fructose intake was reduced from 15% to 4% of daily caloric intake in obese adolescents, a 9-day intervention resulted in decreased levels of D-lactate, which correlated with improved insulin resistance, reduced liver fat, de novo lipogenesis, and dyslipidemia. *: changes in D-lactate in obese subjects; IR: insulin resistance; DNL: de novo lipogenesis. This figure was partly generated using Servier Medical Art, provided by Servier, licensed under a Creative Commons Attribution 3.0 unported license.

As previously mentioned, there is a paucity of studies of MG levels in obesity, MetS, or IR due to the lack of a reliable assay, complex LC-MS/MS methods, and scarce high-purity MG standards. Nevertheless, one indirect measure of MG flow is plasma D-lactate levels, which reflect essentially the end metabolite of MG in vivo. Adult obesity has been linked to elevated serum MG and D-lactate levels. Nevertheless, neither this phenomenon nor the connection between these pathways, fructose metabolism, and childhood obesity has been studied. To shed light on these issues, we conducted a case–control study and an intervention study in teenagers, which we summarize in Figure 4.

First, we contrasted adolescents with obesity and high fructose intake with adolescents of normal weight, as illustrated in the figure [60,61,103]. This cross-sectional study of obese adolescents without overt MetS revealed early proatherogenic changes in lipoprotein profiles, a high prevalence of small dense LDLs (sd-LDLs), and early structural changes in carotid arteries as measured by CIMT and endothelial function when compared to age- and gender-matched lean control subjects. The main conclusions were that (a) obese adolescents had elevated D-lactate levels, a surrogate marker of MG and, thus, triose phosphate fluxes, and (b) there was a strong correlation between D-lactate, LDL size, and sd-LDLs, suggesting that the two derangements associated with early IR may be related either to obesity and IR or to fructose consumption or both.

As previously mentioned, from a mechanistic standpoint, increased MG through fructose-induced triose phosphates (TPs) and fluxes—shared substrates in the pathways of α-GP synthesis, glycolysis, and gluconeogenesis—would most likely lead to elevated D-lactate levels. In proportion to the flux of trioses required to support TG synthesis, MG production increases. In fact, our obese teenagers had much higher TG and TG/HDL-C levels.

In a second investigation, to shed more light on the mechanisms and provide causative evidence of the role of fructose in these pathways, we sought to ascertain if fructose’s higher lipogenic potential and more intense stimulation of MG synthesis could result in greater metabolic disturbance than glucose [88,97,98]. The amount of sugar in the diet of obese adolescents was reduced from 28% to 10% of total calories (fructose thus reduced to 5% of total calories) for nine days without changing the total calories or total carbohydrates. The reduction in sugar calories was compensated with a parallel increase in starch (glucose). D-lactate levels reduced by 50%, and the magnitude of reduction strongly correlated with an improved lipid profile, insulin action, and a reduction in liver fat and DNL. The relevance of this reduction becomes more apparent when we consider that the best study so far on GLO inducers in obesity reduced MG by 37% in 8 weeks (see below, Section 6). If glucose were a substrate equal to fructose for the formation of either MG or DNL, we would not have expected a significant change in either serum D-lactate levels or DNL levels because the participants increased the amount of glucose in their intervention diets since they consumed the same amount of carbohydrates: we decreased fructose while increasing glucose from starch.

Therefore, we proffer that fructose, independent of its caloric equivalent, increases the production of hepatic MG (and thus its detoxified metabolite D-lactate), which in turn contributes considerably to IR and its sequelae, obesity and metabolic syndrome. The study findings emphasize the potential consequences of high fructose intake, especially in adolescents. The intervention, which aimed to reduce fructose consumption and substitute it with glucose from starch, demonstrated a significant impact on metabolic indicators. This suggests that fructose’s unique metabolic pathways, including its role in MG and D-lactate production, contribute more significantly to metabolic disturbances than glucose.

## 6. MG Surges Induced by Fructose: Are They Causative or Bystanders

**Figure 5:**
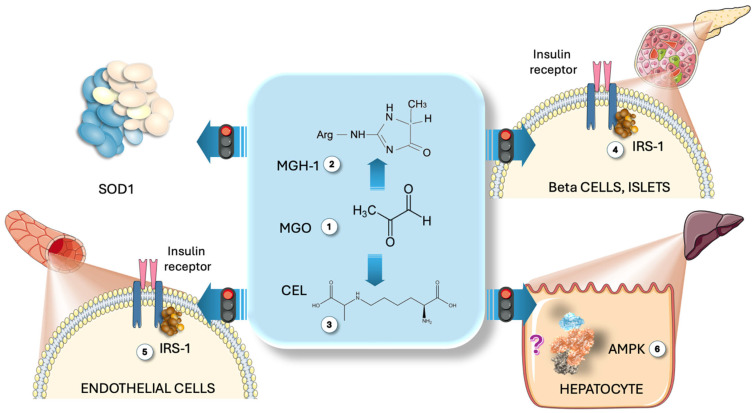
**Direct action of MG on some relevant metabolic pathways.** MG (1) modifies Arg residues to produce MGH-1 (2) and lysine, yielding CEL (3). Preclinical studies indicate that MG alters IRS1 in Langerhans islet beta (4) and endothelial cells (5), disrupting insulin signaling. MG also deactivates SOD, raising ONOO peroxidative stress (3). AMPK, containing Arg and Lys residues in its allosteric sites, may be affected by MG, resulting in a disrupted enzyme response that promotes TG and cholesterol synthesis rather than catabolism (6). MG: methylglyoxal; MGH-1: methylglyoxal-hydroimidazolone 1; CEL: carboxy ethyl lysine; IRS1: insulin receptor substrate 1; AMPK: AMP-activated kinase. This figure was partly generated using Servier Medical Art, provided by Servier, licensed under a Creative Commons Attribution 3.0 unported license.

Whether the associations shown above are the reflection of cause and effect or are simply pointing to a common metabolic node—DHAP excess, which leads to both MG excess and alpha-glycerophosphate accumulation—or other direct actions of fructose metabolism deserves careful further confirmation in future studies. Nevertheless, our data contribute to and support the notion that fructose is an important source of MG, which has not been explored thus far, and deserves further attention. Although much research remains to be conducted, animal studies show that MG itself recapitulates changes akin to IR and diabetes [10]. Regarding putative pathogenic mechanisms, in Figure 5, we summarize some preclinical studies showing that MG modifies IRS1 in beta cells and endothelial cells, leading to disrupted insulin signaling, and that MG inactivates SOD, increasing ONOO peroxidative stress [104,105]. AMPK, a key energy and glucose sensor, has Arg and Lys residues in two of its allosteric sites that may be attacked by MG, leading to a disrupted response of the enzyme. In this case, rather than promoting lipid catabolism (its main function), it would enable TG and cholesterol synthesis [106,107,108,109].

## 7. Glyoxalase Modulators as a Co-Adjuvant/Treatment of the Deleterious Impact of Fructose Metabolism

Previous studies on obesity measuring MG and D-lactate, together with our case–control and intervention results above, suggest that GLO1 modulation should be explored in the context of fructose excess in the diet, which has become pervasive. Ideally, the logical action would be to simply reduce fructose consumption. Unfortunately, this premise has proven to appear all but utopian. Confronted with this reality, GLO1 activation and/or increased expression appears to be a reasonable option to implement.

Studies in humans have shown that lifestyle changes, such as exercise, and small-molecule treatments can increase GLO1 levels to improve metabolic function. Efforts have been made to decrease cellular MG concentrations through clinical strategies after understanding the connection between increased MG formation and vascular complications of diabetes. Early chemical scavenging agents, such as aminoguanidine or phenacylthiazolium bromide, showed effectiveness against MG but were found to be toxic or unstable [110,111,112,113,114,115]. A more effective approach involves increasing the expression and activity of GLO 1. This was achieved through small-molecule activators of the transcription factor Nrf2, which binds to the GLO 1 gene’s antioxidant response element (ARE). The compounds trans-resveratrol (tRES) and hesperetin (HESP) demonstrated pharmacological synergism in activating GLO 1-ARE transcriptional activity [1,2,13,20].

This combination, known as tRES-HESP, showed positive results in preclinical and clinical studies. In endothelial cells, tRES-HESP reduced the expression of receptors for RAGE and cell adhesion molecules, along with a decrease in inflammatory mediator secretion. In fibroblasts and a hepatoblastoma cell line, tRES-HESP increased GSH cellular levels. The combination also proved effective in experimental models, accelerating wound healing in diabetic mice and displaying anti-inflammatory effects [13,20].

There is also clinical evidence from the Healthy Aging Through Functional Food (HATFF) study involving overweight and obese subjects who received tRES-HESP orally for 8 weeks. Specifically, tRES-HESP increased GLO 1 activity in cells by 22%, resulting in a 37% decrease in MG plasma levels. This reduction was associated with improved IR and reduced low-grade inflammation (compared with the 50% reduction in D-lactate and metabolic improvement we found in 1/6th of this time using fructose restriction, shown in [88]). Physiologically, tRES-HESP effectively corrected IR in overweight and obese individuals, restoring insulin sensitivity to levels observed in lean subjects. The combination also demonstrated potential benefits in blood pressure and dyslipidemia. The synergistic effects of tRES-HESP were attributed to the improved bioavailability of tRES facilitated by HESP and pharmacological synergy in Nrf2 activation. In summary, tRES-HESP appears as a promising dietary supplement warranting further clinical assessment.

We suggest that studies should be performed in the context of fructose restriction/overfeeding studies (both preclinical and clinical) to ascertain the potential application of tRES-HESP as a palliative in the context of the current fructose excess and MetS epidemics, especially in younger populations. Its well-tolerated nature and lack of reported adverse effects suggest it could be suitable for this and other chronic and prophylactic treatment applications, prompting ongoing investigations. However, the effectiveness of current NRF2-targeting molecules is relatively modest, but the proof of principle paves the way to finding better agents.

Consequently, there is a pressing need for improved GLO1-targeting strategies due to the complex regulation at transcriptional, post-transcriptional, and post-translational levels. Future research should focus on targeting all these regulatory levels and verifying the importance of GLO1 for metabolic benefits in vivo.

Other molecules, including both nutrients and pharmaceuticals, show apparent effects on GLO1, as reviewed in [23]:

- Isothiocyanates in cruciferous vegetables activate Nrf2, boosting GLO I activity and expression.
- Bardoxolone methyl activates Nrf2-Keap1-ARE, potentially increasing GLO I expression to safeguard kidney function in diabetes.
- Fisetin increased GLO 1 expression and activity, as well as GSH formation, benefiting diabetic patients.
- Mangiferin, a natural xanthone with C-glucoside, prevented diabetic nephropathy by enhancing GLO I function and inhibiting oxidative stress damage and the AGE/RAGE axis.

On the pharmacological side:

- Metformin has long been considered an effective quencher of MG [116,117,118].
- Pyridoxamine induces GLO I activity and is a candidate for treating obesity-related inflammation and preventing diabetic retinopathy. It reduces MG and increases GLO I activity [119,120,121].
- Candesartan, a synthetic drug, stimulates GLO 1, restoring GLO I function and nitric oxide release in cells affected by angiotensin II, improving retinal health in bovines [122].

## 8. Conclusions and Future Avenues

We offer a perspective that complements the current understanding of the role of MG and the glyoxalase cycle in the pathogenesis of insulin resistance and obesity, leading to type 2 DM and CVD. Fructose may be a main substrate providing, in modern times, much of the flux of trioses in the liver to account for a large proportion of MG production. The steady-state MG concentration—and the modification of proteins that may ensue—would then be determined by the flux of trioses, as well as their use in lipogenesis, on the one hand, and their decomposition into MG, on the other, the latter being turned into D-lactate by GLO1 and GLO2. It follows that enhancing the activity and/or expression of GLO1 may be an avenue for the mitigation of the deleterious effect of fructose in the liver. Additional research and validation are needed for these biological pathways. If confirmed, fructose restriction and GLO1 inducers and/or dietary components may cooperate to ease the current metabolic syndrome and MASLD outbreaks.

## Abbreviations

AMPK: AMP-activated protein kinase; ChREBP: carbohydrate-responsive element-binding protein; DHAP: dihydroxy acetone phosphate; DNL: de novo lipogenesis; GLO1 and 2: glyoxalase 1 and 2; GK: glucokinase; HFCS: high-fructose corn syrup; IR: insulin resistance; KHKc: ketohexokinase c; LGSH: s-D-lactoyl glutathione; MASLD: metabolic dysfunction-associated steatotic liver disease; MetS: metabolic syndrome; MODIC: lysine-lysine crosslinks; MOLD: alysine-arginine protein crosslinks; SIRT1: Sirtuin 1; SREBP-1c, sterol regulatory element binding protein 1c; T2DM: type 2 diabetes; TG: triglyceride; tRES-HESP: trans-resveratrol and hesperetin combo; UPR: unfolded protein response; VLDL: very low density lipoprotein.
